# Increased Interleukin-17 and Glucocorticoid Receptor-β Expression in Interstitial Lung Diseases and Corticosteroid Insensitivity

**DOI:** 10.3389/fimmu.2022.905727

**Published:** 2022-07-05

**Authors:** Chun-Yu Lo, Chun-Hua Wang, Chih-Wei Wang, Chih-Jung Chen, Hung-Yu Huang, Fu-Tsai Chung, Yu-Chen Huang, Chang-Wei Lin, Chung-Shu Lee, Chun-Yu Lin, Chiung-Hung Lin, Po-Jui Chang, Ting-Yu Lin, Chih-Chen Heh, Jung-Ru He, Kian Fan Chung

**Affiliations:** ^1^ Department of Thoracic Medicine, Chang Gung Memorial Hospital, Taipei, Taiwan; ^2^ College of Medicine, Chang Gung University, Taoyuan, Taiwan; ^3^ Department of Pathology, Chang Gung Memorial Hospital, Taipei, Taiwan; ^4^ Department of Pathology and Laboratory Medicine, Taichung Veterans General Hospital, Taichung, Taiwan; ^5^ School of Medicine, Chung Shan Medical University, Taichung, Taiwan; ^6^ New Taipei Municipal TuCheng Hospital (Managed by Chang Gung Medical Foundation), New Taipei City, Taiwan; ^7^ Airway Disease Section, National Heart and Lung Institute, Imperial College London and Biomedical Research Unit, Royal Brompton Hospital, London, United Kingdom

**Keywords:** interleukin-17, idiopathic pulmonary fibrosis, sarcoidosis, cryptogenic organizing pneumonia (COP), corticosteroid insensitivity, glucocorticoid receptor-beta

## Abstract

**Background:**

Treatment responsiveness to corticosteroids is excellent for cryptogenic organizing pneumonia (COP) and sarcoidosis, but suboptimal for idiopathic pulmonary fibrosis (IPF)/usual interstitial pneumonia (UIP). We hypothesise that the differential expression of IL-17 contributes to variable corticosteroid sensitivity in different interstitial lung diseases.

**Objective:**

To determine the associations among expression of IL-17, glucocorticoid receptor-β and responsiveness to corticosteroid treatment in interstitial lung diseases.

**Methods:**

Immunohistochemical (IHC) staining was performed on formalin-fixed paraffin-embedded (FFPE) lung tissues obtained by bronchoscopic, CT-guided or surgical biopsies, and quantified by both cell counting (% positive cells) by individuals and by software IHC Profiler plugin of ImageJ (opacity density score). We studied the effect of IL-17 on corticosteroid sensitivity in human fibroblast MRC5 cell line.

**Results:**

Compared with specimens from patients with COP (n =13) and sarcoidosis (n =13), those from IPF patients (n = 21) had greater GR-β and IL-17 expression and neutrophil infiltration. Radiographic progression after oral corticosteroid treatment was positively correlated with the expression in IL-17 and GR-β/GR-α ratio in all patients (COP, sarcoidosis and IPF) and also within the IPF subgroup only. IL-17 expression level was positively associated with GR-β and GR-β/GR-α ratio. In MRC5 cells, exogenous IL-17 increased the production of collagen I and up-regulated GR-β expression and dexamethasone’s suppressive effect on collagen I production was impaired by IL-17, and silencing IL-17 receptor A gene attenuated the effect of IL-17.

**Conclusion:**

Up-regulation of GR-β/GR-α ratio by IL-17 could be associated with the relative corticosteroid-insensitivity of IPF.

## Introduction

Interstitial lung diseases (ILDs) encompass a heterogenous group of non-neoplastic disorders resulting from inflammatory and fibrotic changes of the pulmonary parenchyma and interstitium (1, 2). Idiopathic pulmonary fibrosis (IPF) is a progressive, irreversible fibrosing disease of unknown cause (3). Usual interstitial pneumonia (UIP) is the hallmark finding of IPF, characterized by bilateral subpleural honeycombing and traction bronchiectasis. Fibrotic lung lesions could also develop in a proportion of patients with other types of ILDs (1, 2, 4). Cryptogenic organizing pneumonia (COP) often presents with a relatively short-term illness, with imaging features of perilobular patchy consolidation and ground-glass opacities. Lung biopsies of COP usually show intraluminal organizing fibrosis in distal airspaces, with interstitial inflammatory infiltration and preservation of lung architecture. Pulmonary sarcoidosis can present with constitutional symptoms or be asymptomatic but with hilar and mediastinal lymph node enlargement in the presence or absence of any parenchymal abnormality (4, 5). Sarcoid granulomas are non-necrotizing and usually located along the lymphatic vessels. Whilst IPF often ends up with oxygen desaturation, respiratory failure and even death despite medical treatment, the prognosis and response to corticosteroid therapy of patients with COP and sarcoidosis are usually better (3).

Fibrosis leads to thickening and scarring of the distal bronchiolar airspaces and alveolar walls in subjects with genetic predisposition (6). Environmental factors (smoking, infections, chemicals) injure the alveolar epithelium and promote epithelium-mesenchymal transition and the formation of fibroblastic and myofibroblastic foci, which secrete excessive amounts of extracellular matrix proteins, mainly collagens, resulting in scarring and destruction of the lung architecture. Inflammatory mediators, such as epithelium-derived alarmins, interleukin (IL)-13 and IL-17, are involved in the aberrant healing process (7–9). IL-17 induces neutrophil extracellular traps (10, 11), activates transforming growth factor (TGF)-β signaling, stimulates extracellular matrix and impairs autophagy, which limits epithelial-mesenchymal transition (9, 12, 13). Whether there is any differential IL-17 expression in COP, sarcoidosis and IPF is currently unknown.

Corticosteroids are frequently used for the treatment of ILDs. Glucocorticoids suppress inflammatory responses by reducing inflammatory cell number and pro-inflammatory mediator release, and increase the expression anti-inflammatory cytokines, chemokines, receptors and adhesion molecules (14). At molecular level, glucocorticoids bind to cytosolic glucocorticoid receptor (GR)-α, translocating to the nucleus and binding to the glucocorticoid response elements (GREs) in the promoters of steroid-responsive genes to increase the transcription of anti-inflammatory proteins (trans-activation). The last 50 amino acids of GR-α’s ligand-binding domain are replaced by a non-homologous 15-amino acid sequence in GR-β, an alternatively spliced form of GR. GR-β does not interact with corticosteroids, acting as a negative inhibitor which competes for the binding of GREs, or interferes GR-α translocation (15). Activated GR-α also recruits transcription co-repressor histone deacetylase 2 (HDAC2) to nuclear factor-κB-histone acetyltransferase transcriptome activator complex, silencing activated pro-inflammatory genes (trans-repression) (16). Long-term systemic glucocorticoid treatment could be associated numerous side effects, including adrenal insufficiency and infections (17). Therefore, it is essential to avoid unnecessary glucocorticoid exposure for glucocorticoid-irresponsive ILD patients.

The mechanisms underlying the differential responses to corticosteroid therapy of various ILDs remain unclear. For this reason, we investigated the potential reasons for which IPF is not responsive to high dose corticosteroid therapy, while the other 2 ILDs, sarcoidosis and COP, are. Because lung tissues from IPF patients have been reported to express up-regulated alarmins (thymic stromal lymphopoietin and IL-33) and down-regulated GRα and HDAC2 compared to those from patients with COP and sarcoidosis (8, 18, 19), we hypothesized that increased levels of another alarmin, IL-17, may also contribute to corticosteroid insensitivity in IPF. We therefore investigated the expression of IL-17 and GR-α and β in tissues obtained from these 3 ILDs.

## Materials and Methods

### Subjects

The medical records and high-resolution computed tomographic images of patients in Chang Gung Memorial Hospital, Linkou (a university-based tertiary referral medical center in Taiwan) with tissue-proved cryptogenic organizing pneumonia (ICD 9-CM: 516.36; ICD-10-CM: J84.116), sarcoidosis (ICD 9-CM: 135/517.8; ICD-10-CM: D86.0, D86.2) and idiopathic pulmonary fibrosis (ICD 9-CM: 516.31; ICD-10-CM: J84.112) were reviewed for potential inclusion by chest specialist physicians. Subjects were identified and classified according to guidelines (2, 5). Patients exhibiting any evidence of underlying collagen vascular diseases or lung infections were excluded. Formalin-fixed paraffin-embedded (FFPE) lung specimens were previously obtained by surgeries, bronchoscopic biopsies or CT-guided biopsies between January 1999 and January 2020. Specimens from subjects who had been treated with prednisolone at a cumulative dose of ≥ 1 g or equivalent within 3 continuous months were obtained from the Tissue Bank of Chang Gung Memorial Hospital, Linkou. A waiver of inform consent requirement was approved by the Institutional Review Board of Chang Gung Memorial Hospital due to the retrospective nature of the study and complete unidentification of the samples (IRB number: 202001538B0).

### Histopathological Evaluation

The sections of FFPE lung specimens stained with hematoxylin and eosin (H&E), as well as acid fast and Gömöri methenamine silver to rule out microorganisms and fungi as needed, were reviewed by two independent pathologists under light microscopy (1, 2, 5). The diagnosis of COP was made on the basis of demonstration of plugs of loose connective tissue within the bronchioles, alveolar ducts and spaces. Sarcoidosis was diagnosed by the presence of non-caseating granulomas in a lymphatic distribution. IPF was recognized by the pathologic feature of the UIP pattern (architectural destruction, fibrosis with honeycombing, scattered fibroblastic foci in a patchy distribution with involvement of the periphery of the acinus or lobule). The number of neutrophils per high power field (HPF, average of three randomly selected area) stained by H&E was enumerated by a blinded observer with a light microscopy at x400 magnification (BX51, Olympus, Tokyo, Japan).

### Chest Radiograph Grading of Anatomical Extent of Disease

Posteroanterior chest radiographs before and after oral corticosteroid therapy (OCS) were assessed by two independent chest physicians, and they arrived at an agreed grading for each patient. A grading of the extent of disease proposed by the World Health Organization (1960) was adopted (20): 0 = no involvement; 1 = trivial, minimal lesions regarded as inactive; 2 = slight, minimal or rather larger lesion regarded as active; 3 = limited, lesions of greater extent, involving a total area of lung less than the right upper lobe; 4 = moderate, lesions of greater extent than in 3 but whose total extent, even if bilateral, not exceeding an area equivalent to the whole of one lung; 5 = extensive, lesions involved an area of more than the whole of one lung; 6 = gross, very extensive bilateral diseases. Response to therapy was determined by change from baseline in chest radiographic grading of the extent of disease after oral corticosteroids (post-OCS CXR CFB).

### Immunohistochemical (IHC) Staining

IHC staining of FFPE lung specimens was performed using IHC Select^®^ HRP Detection Set (DET-HP1000, Millipore, Burlington, MA) to determine the expression and distribution of IL-17 and transcriptional factors. Briefly, 4 μm thick sections were dewaxed and rehydrated in graded alcohol series. The sections were washed in distilled water, boiled in a microwave oven for epitope retrieval in sodium citrate buffer (10mM) at pH 6.0. The slides were treated with 3% H_2_O_2_ to inhibit endogenous peroxidase, followed by 3,3’-Diaminobenzidine (DAB) rinse buffer, equilibrated in PBS and blocking solution provided in the DAB IHC Select kit (DAB500, Millipore, Burlington, MA). The slides were immunostained with primary rabbit anti-GR-α (ab3580, Abcam, Cambridge, UK), anti-GR-β (ab233165, Abcam, Cambridge, UK), anti-HDAC2 (ab32117, Abcam, Cambridge, UK), anti-IL-17_A_ (sc-7927, Santa Cruz, Dallas, TX) and anti-IL-17RA (NBP2-25258, Novus Biologicals, Centennial, CO) antibodies, and incubated overnight at 4°C. The primary antibodies were omitted to serve as technical negative control and appropriate positive control tissues were used. The slides were washed and incubated with secondary antibodies. Nuclei were counterstained blue with Mayer’s hematoxylin (HMM125, ScyTek, Logan, UT), dehydrated, and mounted with Permount.

The results of IHC staining were evaluated by physician counting and software ImageJ (National Institutes of Health, Bethesda, Maryland). Each component immunoreactivity was measured on three different representative slide areas (×400 magnification; area equals 0.44 mm^2^ per field) randomly selected using a light microscope (BX51, Olympus, Tokyo, Japan). The percentage of cell marker-positive nucleated cells of total nucleated cells was calculated and expressed as cells/100 nucleated cells by two independent physicians blinded for clinical information and the percentage of cell marker-positive nucleated cells of total nucleated cells was calculated and expressed as cells/100 nucleated cells. The IHC staining results were also quantified automatically with IHC Profiler plugin of ImageJ and further divided into four groups based on the number of positive cells: high positive, positive, low positive and negative (21). The following algebraic formula was used to calculate the IHC optical density score (from 1 to 4) for the IHC images. IHC optical density score (OD) = (percentage contribution of high positive ×4+ percentage contribution of positive ×3+ percentage contribution of weak positive ×2+ percentage contribution of negative ×1)/100 (22).

### Fibroblast Culture and Small Interference (Si) RNA Silencing of IL-17 Receptor A (IL-17RA)

5 × 10^5^ Fibroblast MRC5 cells (CCL-171, ATCC, Manassas, VA) were plated and grown overnight for 24 h in 2.5mL of Eagle’s minimum essential medium (#30-2003, ATCC, Manassas, VA) supplemented with 20% fetal bovine serum. Cells were serum-starved for 24h, transfected with either Si-IL-17R_A_ RNA (#s522428, Ambion, Austin, TX) or scrambled control siRNA (Si-Ctrl, #4390843, Ambion, Austin, TX) using Lipofectamine RNAwereiMAX reagent (Invitrogen, Waltham, MA) following the manufacturer’s instructions for another 24h, stimulated with recombinant human IL-17 (#317-ILB, R&D, Minneapolis, MN) and dexamethasone (D8893, Sigma-Aldrich, St. Louis, MO) and were then harvested to determine the levels of mRNA and protein.

### Quantitative Real-Time PCR (qRT-PCR)

Total RNA was isolated from MRC5 cells by using the TRIzol reagent (#15596018, Ambion, Austin, TX) and reverse-transcribed with High Capacity cDNA Reverse Transcription Kit (#4374967, Appliedbiosystems, Foster City, CA). Quantitative real-time PCR (qRT-PCR) was performed using Rotor-Gene SYBR Green PCR Kit (#204074, Qiagen, Hilden, Germany) on MyGo Pro instrument (IT-IS Life Science, Middlesbrough, UK). The sequences of the primers used were as follows: GR-α forward 5’-GCA GTG GAA GGA CAG CAC AA and reverse 5’-TCC TGT AGT GGC CTG CTG AA-3’; GR-β forward 5’-CCT AAG GAC GGT CTG AAG AGC-3’ and reverse 5’-CCA CGT ATC CTA AAA GGG CAC-3’; HDAC2 forward 5’- GGG AAT ACT TTC CTG GCA CA-3’and reverse 5’- ACG GAT TGT GTA GCC ACC TC-3’; collagen I forward 5’-CCT CAA GGG CTC CAA CGA G-3’ and reverse 5’-TCA ATC ACT GTC TTG CCC CA-3’; 18s forward 5’-CTT AGA GGG ACA AGT GGC G-3’ and reverse 5’-AGC CTG AGC CAG TCA GTG TA-3’. Melting curve analysis was carried out to ensure the presence of each specific PCR product.

### Western Blotting

Whole cell protein was extracted using home-made radioimmunoprecipitation assay buffer (50mM Tris-HCl(pH7.4), 150mM Sodium chloride, 1% NP-40, 0.5% Sodium deoxycholate, 0.1% SDS). Protein concentration was measured using Bio-Rad Protein Assay Dye Reagent Concentrate (#500-0006, Bio-Rad, Hercules, CA) and proteins were preserved at −20°C. 50–100 μg protein samples were injected into wells of SDS–PAGE gels, separated using electrophoresis and transferred to membranes. Membrane-bound proteins were identified using rabbit antibodies for anti-collagen I (#72026, Cell signaling, Danvers, MA), anti-GR-α (#12041, Cell signaling, Danvers, MA), anti-GR-β (ab3581, Abcam, Cambridge, UK), anti-HDAC2 (#57156, Cell signaling, Danvers, MA), followed by anti-rabbit–horseradish peroxidase antibody. β-Tubulin (#15115, Cell signaling, Danvers, MA) or Actin (#Mab1501, Millipore, Burlington, MA) were used as controls.

### Statistical Analysis

Data are presented as mean ± standard error of the mean (SEM). Statistical analysis was carried out using the GraphPad Prism v.7 software package (GraphPad Prism Software Inc, San Diego, California, USA). The differences between disease groups were determined by Kruskal-Wallis test, followed by Dunn’s *post-hoc* test for more than three groups. Intra-group comparisons of two and more than two conditions were carried out using Wilcoxon test and the Friedman test followed by Dunn’s *post-hoc* test respectively. Correlations were determined by Spearman’s rank correlation. *P* value < 0.05 was considered as statistically significant.

## Results

The clinical presentation and staining results of the ILD subjects are shown in **Table 1**. The cumulative prednisolone-equivalent exposures were not statistically different among three disease groups. After OCS treatment, IPF patients showed deterioration in chest radiographic disease extent grading, while patients with COP and sarcoidosis showed improvement. IPF patient also showed less improvement in forced vital capacity compared to sarcoidosis patients. The cumulative prednisolone dose was not corelated with the extent of radiographic changes (ILD: r_s_ = 0.14, p = 0.38; IPF subgroup: r_s_ = 0.42, p = 0.07), so it is unlikely that the rapid deterioration shown on chest radiographs in IPF was due to lower dose of corticosteroid treatment. Thirteen IPF patients had received anti-fibrotic agents during oral corticosteroid treatment (nintedanib: 9; pirfenidone: 4; nintedanib and pirfenidone: 1) and nine IPF patients eventually underwent lung transplantation.

**Table 1:** Patient characteristics and immunohistochemistry results.

	COP	Sarcoidosis	IPF
	n = 13	n = 13	n = 21
Patient characteristics			
Age, years	57.7 ± 4.8	49.2 ± 3.8**	63.3 ± 1.7
Gender, Male/female	4/9	4/9	16/5
Cumulative OCS (mg)	739.2 ± 138.7	990.8 ± 214.1	895.5 ± 147.3
CXR	2.84 ± 0.22	2.54 ± 0.31	3.23 ± 0.18
Post-OCS CXR CFB	-0.31 ± 0.31*	-0.69 ± 0.32**	1.00 ± 0.27
FVC (L)	1.553 ± 0.138*	2.373 ± 0.234	1.713 ± 0.156
Post-OCS FVC (L) CFB	0.230 ± 0.074	0.368 ± 0.216*	-0.208 ± 0.085
FVC %pred	55.9 ± 4.2	73.2 ± 5.2	65.7 ± 4.6
Post-OCS FVC %pred CFB	7.9 ± 2.9	5.6 ± 6.1	-7.3 ± 6.2
Staining findings			
Neutrophils/HPF	0.85 ± 0.23*	0.85 ± 0.19*	2.97± 0.74
%IL-17	46.5 ± 4.4*	46.7 ± 5.2*	63.0 ± 3.4
IL-17 OD	1.32± 0.04*	1.31 ± 0.05**	1.48 ± 0.04
%IL-17RA	47.3 ± 6.2	45.4 ± 5.2	59.2 ± 4.1
IL-17RA OD	1.44 ± 0.03	1.41 ± 0.03	1.44 ± 0.03
%GR-β	55.7 ± 3.8****	58.2 ± 3.7***	73.8 ± 1.0
GR-β OD	1.33 ± 0.02*	1.35 ± 0.05	1.44 ± 0.02
%GR-α	65.9 ± 3.4	58.8 ± 4.9	60.2 ± 3.4
GR-α OD	1.33 ± 0.01	1.33 ± 0.02	1.33 ± 0.01
%GR-β/%GR-α	0.88 ± 0.08**	1.04 ± 0.08	1.33 ± 0.09
GR-β OD/GR-α OD	1.00 ± 0.02*	1.01 ± 0.02	1.09 ± 0.02
%HDAC2	55.8 ± 7.0	52.2 ± 6.6	57.6 ± 3.0
HDAC2 OD	1.30 ± 0.05	1.29 ± 0.02	1.29 ± 0.04

Data are present as mean ± SEM. COP, cryptogenic organizing pneumonia; IPF, idiopathic pulmonary fibrosis; CXR, chest radiographic grading of the extent of disease; CFB, change from baseline; OCS, oral corticosteroids; cumulative OCS, cumulative prednisolone or equivalent within 3 continuous months; FVC, forced vital capacity; %pred, percent of predicted value; HPF, high power fields (magnification, x400); IL-17, interleukin-17; IL-17RA, interleukin-17 receptor A; GR, glucocorticoid receptor; HDAC2, histone deacetylase 2; OD, opacity density. *P < 0.05, **P < 0.01, ***P < 0.001, ****P < 0.0001 versus IPF.

**Figure 1:**
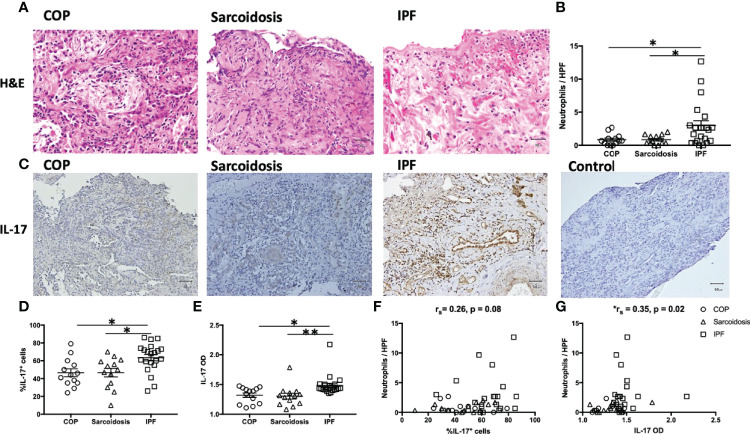
Increased neutrophil infiltration and more IL-17 expression in IPF lungs. **(A)** Representative H&E images of lung specimens from subjects with COP, sarcoidosis and IPF. Magnification ×400. **(B)** IPF lungs exhibited greater number of neutrophils. **(C)** Representative immunohistochemical image of lung specimens from subjects with COP, sarcoidosis and IPF. Magnification ×200. **(D, E)** Immunohistochemical staining showed IPF lungs had a greater percentage of IL-17^+^ cells and higher IL-17 OD. **(F, G)** The expression of IL-17 is positively correlated with neutrophil counts in lung specimens. IL-17, interleukin-17; IPF, idiopathic pulmonary fibrosis; COP, cryptogenic organizing pneumonia; H&E, hematoxylin and eosin; HPF, high power fields (magnification, x400); OD, opacity density score; *p < 0.05; **p < 0.01.

**Figure 2:**
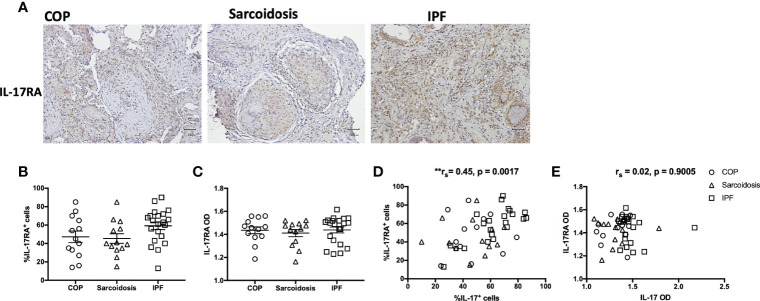
IL-17RA expression in ILD lungs. **(A)** Representative immunohistochemical image of lung specimens from subjects with COP, sarcoidosis and IPF. Magnification ×200. **(B, C)** Immunohistochemical staining showed no significant difference in IL-17RA expression amongst lung specimens from patients with COP, sarcoidosis and IPF. **(D, E)** The correlation between IL-17RA expression and IL-17 expression in ILDs. IL-17, interleukin-17; IL-17RA, interleukin-17 receptor A; IPF, idiopathic pulmonary fibrosis; COP, cryptogenic organizing pneumonia; OD, opacity density score; **p < 0.01.

Neutrophils and IL-17 have been associated with the development of lung fibrosis in animal models (23, 24). An increased number of neutrophils in H&E staining was found in IPF lungs compared with COP lungs and sarcoidosis lungs (**Figures 1A, B** and **Table 1**). Using IHC staining (**Figure 1C**), we found a higher IL-17 expression in lung tissues from IPF patients in terms of both examiners’ determined proportion and software-determined OD compared with that in COP lungs and sarcoidosis lungs (**Figures 1D, E**). IL-17 OD was positively correlated with the neutrophil numbers in tissue (**Figure 1G** and **Table 2**). The expression of IL-17RA was not significantly different amongst specimens from three disease groups (**Figures 2A–C**). %IL-17RA+ cells was positively correlated with %IL-17+ cells (**Figure 2D** and **Table 2**). However, IL-17RA expression was not correlated with the extent of radiographic deterioration (**Table 3**).

**Table 2:** Correlations between IL-17 and staining findings (neutrophil counts, IL-17, GR-β, GR-α, GR-β/GR-α, HDAC2).

	%IL-17	p value		IL-17 OD	p value
ILD					
Neutrophils/HPF	0.26	0.0752	Neutrophils/HPF	0.35*	0.0157
%IL-17RA	0.45**	0.0017	IL-17 OD	0.02	0.9005
%GR-β	0.39**	0.0065	GR-β OD	0.61****	< 0.0001
%GR-α	-0.00	0.9903	GR-α OD	-0.08	0.5758
%GR-β/%GR-α	0.29	0.0510	GR-β OD/GR-α OD	0.64****	< 0.0001
%HDAC2	0.00	0.9858	HDAC2 OD	-0.09	0.5455
IPF					
Neutrophils/HPF	0.12	0.6160	Neutrophils/HPF	0.17	0.45
%IL-17RA	0.63**	0.0023	IL-17 OD	-0.27	0.2294
%GR-β	0.12	0.6080	GR-β OD	-0.03	0.8933
%GR-α	0.28	0.2235	GR-α OD	-0.5948**	0.0045
%GR-β/%GR-α	-0.18	0.4419	GR-β OD/GR-α OD	0.50*	0.0206
%HDAC2	0.18	0.4437	HDAC2 OD	-0.16	0.4927

Correlations were determined by Spearman’s rank correlation. HPF, high power fields (magnification, x400); IL-17, interleukin-17; IL-17RA, interleukin-17 receptor A; GR, glucocorticoid receptor; HDAC2, histone deacetylase 2; OD, opacity density score; *p < 0.05; **p < 0.01; ****P < 0.0001.

**Table 3:** Correlations between radiographic deterioration and staining findings (neutrophil counts, IL-17, GR-β, GR-α, GR-β/GR-α, HDAC2) in ILDs (COP, sarcoidosis and IPF in combination) and IPF subgroup.

Radiograph grading CFB after OCS	ILD	p value	IPF	p value
Neutrophils/HPF	0.12	0.3917	-0.10	0.67
%IL-17	0.39**	0.0067	0.44*	0.0462
IL-17 OD	0.48***	0.0006	0.72***	0.0003
%IL-17RA	0.02	0.9153	-0.2	0.4285
IL-17RA OD	0.02	0.8917	-0.08	0.7409
%GR-β	0.46**	0.0012	0.32	0.1528
GR-β OD	0.50***	0.0003	0.38	0.0904
%GR-α	0.05	0.7178	0.06	0.8041
GR-α OD	-0.20	0.1890	-0.63**	0.0021
%GR-β/%GR-α	0.23	0.1146	0.09	0.7135
GR-β OD/GR-α OD	0.57****	< 0.0001	0.84****	< 0.0001
%HDAC2	0.01	0.9547	-0.11	0.6426
HDAC2 OD	-0.25	0.0916	-0.28	0.2112

CXR, chest radiographic grading of the extent of disease; CFB, change from baseline; OCS, oral corticosteroids; ILD, interstitial lung diseases; COP, cryptogenic organizing pneumonia; IPF, idiopathic pulmonary fibrosis; HPF, high power fields (magnification, x400); IL-17, interleukin-17; IL-17RA, interleukin-17 receptor A; GR, glucocorticoid receptor; HDAC2, histone deacetylase 2; OD, opacity density score; *p < 0.05; **p < 0.01; ***p < 0.001; ****P < 0.0001 (Spearman’s rank correlation).

**Figure 3:**
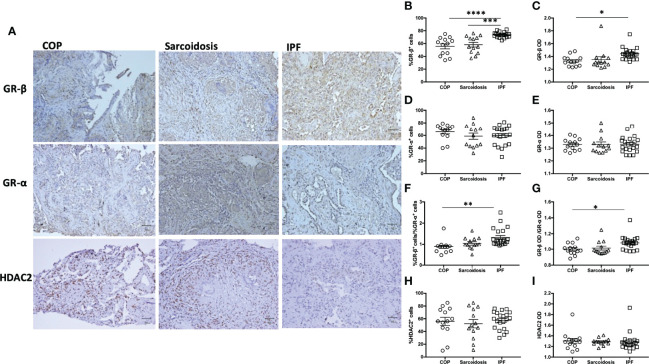
Greater expression of GR-β in IPF. **(A)** Representative immunohistochemical stain images of lung specimens from subjects with COP, sarcoidosis and IPF. Magnification X200. Proportion of positive staining cells **(B, D, F, H)** and OD **(C, E, G, I)** in lung specimens from subjects with ILDs (COP, sarcoidosis and IPF in combination), determined by both examiners and software-determined opacity density score. IPF patients had greater GR-β and GR-β/GR-α ratio. Horizontal lines represent the means ± SEM values for each group. GR, glucocorticoid receptor; IL-17, interleukin-17; IPF, idiopathic pulmonary fibrosis; COP, cryptogenic organizing pneumonia; OD, opacity density score; *p < 0.05; **p < 0.01, ***p < 0.001, ****p < 0.0001.

Since GR-β antagonizes corticosteroid-triggered signaling through GR-α and its upregulation is associated with glucocorticoid insensitivity (25), IHC for GR-α and GR-β and HDAC2 was performed (**Figure 3A**). 73.8 ± 1.0% of cells in IPF specimens were positive for GR-β expression, greater than the cells in the lung biopsies of patients with COP (55.7 ± 3.8%, p < 0.0001) and sarcoidosis (58.2 ± 3.7%, p < 0.001) (**Figure 3B**). Automated quantification also showed that IPF had a higher GR-β OD compared with COP. %GR-β/%GR-α and GR-β OD/GR-α OD ratios were higher in IPF samples compared to the ratios measured in COP (**Figure 3C**). The expression of GR-α (**Figures 3D, E**) and HDAC2 (**Figures 3H, I**) were not significantly different amongst specimens from three disease groups. We did not find correlations between cumulative prednisolone exposure and the expression of GR-α (%GR-α: r_s_ = 0.04, p = 0.81; GR-α OD: r_s_ = 0.02, p = 0.90), GR-β (%GR-β: r_s_ = 0.08, p = 0.58; GR-β OD: r_s_ = -0.22, p = 0.14), HDAC2 (%HDAC2: r_s_ = -0.09, p = 0.54; HDAC2 OD: r_s_ = -0.04, p = 0.78), IL-17 (%IL-17: r_s_ = -0.20, p = 0.17; IL-17 OD: r_s_ = -0.16, p = 0.29), IL-17RA (%IL-17RA: r_s_ = -0.06, p = 0.68; IL-17RA OD: r_s_ = 0.08, p = 0.58) or neutrophil counts (-0.08, p = 0.60).

The radiographic progression of lung fibrosis of our ILD population (COP, sarcoidosis and IPF in combination) and after oral corticosteroid (post-OCS CXR CFB) was positively associated with IL-17 expression and GR-β OD/GR-α OD ratio, as well as GR-β expression (**Table 3**). In the IPF subgroup, CXR CFB was also positively correlated with the expression of IL-17 and GR-β OD/GR-α OD ratio. Interestingly, post-corticosteroid CXR CFB was inversely associated with GR-α OD but not correlated with GR-β expression.

**Figure 4:**
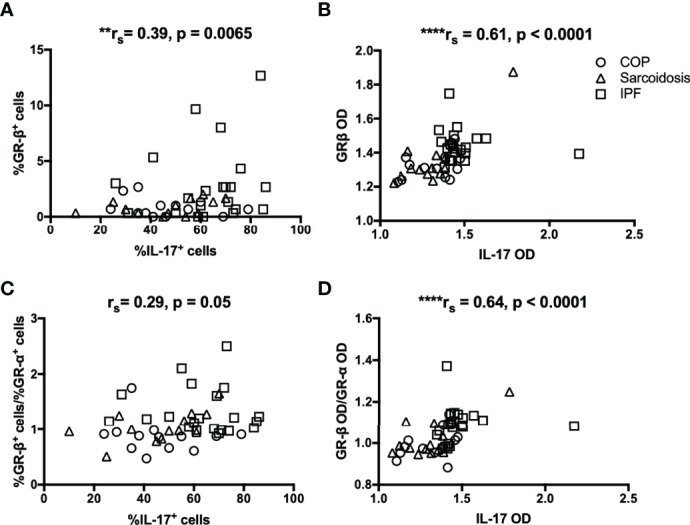
The relationship between IL-17 and GR isoforms in ILDs. **(A–D)** Immunohistochemical staining showed IL-17 expression was positively associated with GR-β and GR-β/GR-α ratio in ILD lungs (COP, sarcoidosis and IPF in combination). IL-17, interleukin; ILD, interstitial lung disease; IPF, idiopathic pulmonary fibrosis; COP, cryptogenic organizing pneumonia; GR, glucocorticoid receptor; HDAC2, histone deacetylase 2; OD, opacity density score. **p < 0.01, ****p < 0.0001.

Since we observed a greater expression of IL-17 in IPF compared with corticosteroid-sensitive COP and sarcoidosis, we further investigated the relationship between expression of IL-17 and transcriptional factors associated with corticosteroid action. IHC data showed correlations between IL-17 and GR-β/GR-α ratio in both our ILD subjects and IPF subgroup (**Figures 4A, B** and **Table 2**). However, IL-17 expression was positively associated with GR-β in whole ILD population (**Figure 4D**) and inversely associated with GR-α in the IPF group.

**Figure 5:**
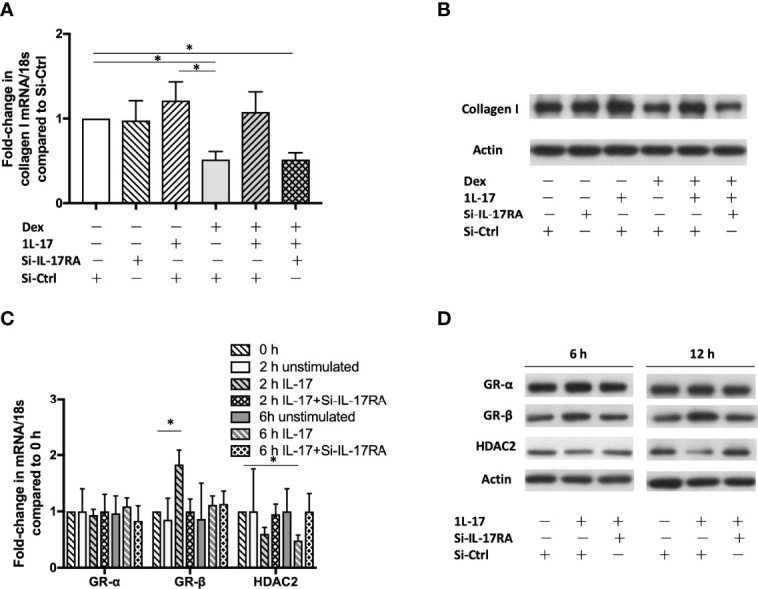
GR-β up-regulation, HDAC2 down-regulation and corticosteroid insensitivity induced by IL-17. Human fibroblast MRC5 cells were transfected with Si-IL-17RA or scrambled Si-Ctrl and then incubated with IL-17 (10ng/mL). mRNA was determined by RT-qPCR and protein was determined by western blotting. **(A, B)** Dexamethasone (10^-7^ M) reduces collagen I production at both mRNA level (2h) and protein level (6h), but the suppressive effect was hampered by IL-17. Silencing IL-17RA gene restored dexamethasone’s suppressive effect. The GR-β mRNA expression was up-regulated at 2h **(C)** and protein was increased at 6h and 12h **(D)**. IL-17 also down-regulated HDAC2 at mRNA and protein level. Silencing IL-17RA gene attenuated IL-17’s effect on GR-β up-regulation and HDAC2 down-regulation. Bars represent means ± SEM of 6 experiments. Si, small interfering RNA; Ctrl, control; IL, interleukin; IL-17RA, interleukin-17 receptor A; Dex, dexamethasone; GR, glucocorticoid receptor; HDAC2, histone deacetylase 2. *p < 0.05.

To confirm the role of IL-17 in the development of corticosteroid insensitivity, fibroblast MRC5 (a human fibroblast cell line derived from normal lung tissue) were plated, grown overnight, serum starved, transfected with Si-IL-17RA or Si-Ctrl and then stimulated with IL-17A in the absence or presence of dexamethasone (10^-7^ M). Dexamethasone’s suppressive effect on production of collagen I was compromised at both mRNA and protein level, determined by qRT-PCR (2h, n = 6) and western blotting (6h), respectively (**Figures 5A, B**). Silencing IL-17RA gene restored dexamethasone’s suppressive effect. In the presence of exogenous IL-17 (10ng/mL), dexamethasone’s suppressive effect was compromised. We also observed that IL-17 (10ng/mL) also up-regulated GR-β (mRNA 2h, protein 6h and 12h) and down-regulated HDAC2 (mRNA 6h, protein 12h, **Figures 5C, D**). The effect of IL-17 on GR-β up-regulation and HDAC2 down-regulation could be attenuated by silencing IL-17RA.

## Discussion

In the present study, we have shown that in lung tissues from IPF patients, there was greater expression of IL-17 with up-regulated GR-β level and increased neutrophil infiltration compared to tissues from sarcoidosis and COP patients However, GR-α and HDAC2 expression were not different. These results provide some mechanism as to why IPF patients may not respond to corticosteroid therapy compared to the patients suffering from the other 2 ILD’s. Furthermore, our experiments done on fibroblasts show that IL-17 can up-regulate GR-β and down-regulated HDAC2, which might underlie the way in which IL-17 induces corticosteroid insensitivity.

IPF specimens exhibited a stronger IL-17 expression and an increased neutrophil infiltration. IL-17 is generated by type 17 CD4+ T-helper (Th17) cells, CD8+ T cells, γδ T-cells, natural killer T cells and type 3 innate lymphoid cells (ILC3) (26). IL-17 acts through IL-17R_A_/1L-17R_C_ heterodimer, triggering signals mediated by adaptor protein CIKS, TNFR-associated factor 6 and nuclear factor-κB (27), leading to the transcription of Th17 cytokine- responsive genes. IL-17 facilitates the release of IL-6 and IL-8 from structural cells and augments neutrophilia (28). Monocyte and neutrophil levels were positively associated with progression from ‘indeterminate for usual interstitial pneumonia’ to IPF (29). A bronchoalveolar lavage (BAL) lower neutrophil-to-lymphocyte ratio has been demonstrated in sarcoidosis and chronic hypersensitivity pneumonitis compared to IPF patients (30). BAL and biopsies from IPF patients also showed a greater amount of IL-17_A_ compared with those from normal volunteers, and the pro-fibrotic effect of IL-17 appears to be IL-1- and TGF-β-dependent (24). IL-17 has been shown to play a direct role in causing fibroblast proliferation, generation of extracellular and myofibroblast differentiation from normal and IPF lungs (31). Intriguingly, although sarcoidosis has been also viewed as a T_H_17 cell-associated disease (32), the IPF specimens showed even stronger IL-17 expression compared with specimens from sarcoidosis subjects. Anti-IL-17 antibody attenuated the severity of lung fibrosis in bleomycin-treated mice (23). Monoclonal antibodies against IL-17 and IL-17 receptor are under development for psoriasis and arthritis. It is worth investigating the effectiveness of these biologics for human progressive fibrosing interstitial lung diseases.

The responses to corticosteroid treatment in ILD patients are diverse: excellent in COP, fair in sarcoidosis but poor in IPF (33). Although oral prednisolone combined with azathioprine and N-acetylcysteine increased mortality and hospitalization in IPF patients (34), administration of systemic corticosteroids was still recommended for acute exacerbation of IPF by the ATS/ERS/JRS/ALAT evidence-based guideline (35). OCS are now standard first-line treatment for sarcoidosis patients with respiratory symptoms and progressive radiographical abnormalities (33, 36). For COP patients with progressive symptoms and impaired lung function, oral prednisolone yielded a 60% complete response rate and 27% partial response rate (33). Corticosteroid insensitivity is associated with reduced GR-α expression, increased GR-α phosphorylation by means of activation of p38 mitogen-activated protein kinase, reduced expression of histone deacetylase (HDAC)2 and increased expression of GR-β which competes GR-α (25). GR-β, a splicing variant of GR-α, fails to bind corticosteroid or to activate gene expression (15). GR-β is associated with corticosteroid-insensitive asthma and ulcerative colitis (37, 38). Higher GR-α expression and lower HDAC2 expression in patients with sarcoidosis and COP compared with those in IPF patients has been previously reported (18, 19), and we highlight the importance of GR-β/GR-α in corticosteroid-insensitive IPF. Although reduced HDAC2 is associated with corticosteroid insensitivity in smokers and patients with severe asthma, chronic obstructive pulmonary disease, nephrotic syndrome and Sudden Sensorineural Hearing Loss insensitive to corticosteroid treatment (16, 39, 40), we did not find a differential HDAC2 expression in corticosteroid-sensitive and corticosteroid-insensitive ILDs. HDACs had a strong induction in IPF compared with normal subjects (41). The role of HDAC2 inhibition in the treatment of lung fibrosis is still to be determined.

IL-17 may hamper the suppressive effect of corticosteroids on the production of ECM in lung fibrosis. The excess of T_H_17 cytokines is blamed for the neutrophilic inflammation in T_H_2-low severe asthma (42). Dexamethasone promotes the differentiation of T_H_17 cells *in vitro*, and failed to ameliorate neutrophilia *in vivo* (43). The corticosteroid insensitivity in obese asthmatic patients can be attributed to the IL-17-downregulated GR-α/GR-β ratio (44). GR-β is disproportionately induced by tumour necrosis factor-α, IL-1, IL-17 and staphylococcal enterotoxin (45–47). IL-17 hampers dexamethasone’s inhibitory effect on IL-6 expression in PBMCs by increasing GR-β (48), and budesonide’s suppressive effect on IL-8 production in airway epithelium 16HBE cells through reduction of HDAC2 activity (49). We also observed a positive correlation between IL-17 and GR-β/GR-α in ILD specimens, and a reduction of GRβ and HDAC2 in IL-17-treated fibroblasts. These data indicate that IL-17 may be related to corticosteroid insensitivity in IPF.

The results of our study may have been influenced by limitations of retrospective study design. The baseline characteristics of three disease groups could not be well-matched in the retrospective study design setting. The duration and dosage of prednisolone treatment given to ILD patients might have not been enough to show therapeutic effect. Since biopsy is often done to provide diagnosis which could not be confirmed by less invasive procedures, the clinical presentations of our ILD patients might not be the most typical ones in the clinical setting. Secondly, we quantified the results by counting the proportion of IHC staining positive cells, which might not fully reflect the intensity of cytokine expression in extracellular matrix. Thirdly, we did not identify the cell types that demonstrated increased expression of IL-17 and reduced expression of GR-β in the biopsies/lung tissues obtained from these patients with ILDs. Fourthly, although enlarged hilar/mediastinal lymph node is the signature imaging feature of pulmonary sarcoidosis, but we mainly looked at the specimens obtained from lungs fields with granuloma. It might also be helpful to compare the effect of IL-17 treatment on IPF lung fibroblast cell lines, such as CC-7231 or CSC-C8082L-IPF, with the normal lung fibroblast MRC5 cell line. It is also worth investigating whether anti-IL-17 or anti-IL-17R biologics and corticosteroids in combination could slower the progression of IPF in the clinical setting to clarify the causal or mediation relationships amongst IL-17, GR-β and response to corticosteroids.

Although it has been recognized that corticosteroids may not be helpful for IPF patients, the mechanisms of corticosteroid insensitivity in IPF have not been previously published. Our findings indicate a role for IL-17 in modulating the ratio of GR-β/GR-α that may lead to the mechanism of corticosteroid insensitivity in IPF. Therefore, IL-17 could be a potential therapeutic target for ILD patients with poor response to corticosteroid treatment. Not only will an anti-IL17 antibody approach would curtail the effect of IL-17 on the process of fibrosis, this will also prevent the effect of IL-17 in inhibiting the therapeutic effects of corticosteroids. Thus, a combination of corticosteroid therapy with an anti-IL-17 approach would be very promising in the treatment of ILDs, in particular IPF.

## Data Availability Statement

The raw data supporting the conclusions of this article will be made available by the authors, without undue reservation.

## Ethics Statement

The studies involving human participants were reviewed and approved by Institutional Review Board of Chang Gung Memorial Hospital. Written informed consent for participation was not required for this study in accordance with the national legislation and the institutional requirements.

## Author Contributions

C-YLo designed the experiments. C-WW and C-JC reviewed the lung tissue specimens. C-CH did immunohistochemical staining. J-RH did polymerase chain reaction and western blotting. H-YH, F-TC, Y-CH, C-WL, C-SL, C-YLin, C-HL, and T-YL reviewed the medical records and radiographic images, quantified IHC results, P-JC and KC participated in the interpretation of the data and discussed the findings. C-YLo and KC finalized the manuscript. All authors contributed to the article and approved the submitted version.

## Funding

This work was supported by the Ministry of Science and Technology of Taiwan (grant number 110-2314-B-182A-142-) and Chang Gung Memorial Hospital Research Project (grant number CMRPG1K0161).

## Conflict of Interest

The authors declare that the research was conducted in the absence of any commercial or financial relationships that could be construed as a potential conflict of interest.

## Publisher’s Note

All claims expressed in this article are solely those of the authors and do not necessarily represent those of their affiliated organizations, or those of the publisher, the editors and the reviewers. Any product that may be evaluated in this article, or claim that may be made by its manufacturer, is not guaranteed or endorsed by the publisher.

## Abbreviations

ILD, interstitial lung diseases; COP, cryptogenic organizing pneumonia; IPF, idiopathic pulmonary fibrosis; UIP, Usual interstitial pneumonia; CXR, chest radiographic grading of the extent of disease; CFB, change from baseline; OCS, oral corticosteroids; H&E, hematoxylin and eosin; IHC, immunohistochemical; Formalin-fixed paraffin-embedded (FFPE); HPF, high power fields (magnification, x400); IL-17, interleukin-17; GR, glucocorticoid receptor; HDAC2, histone deacetylase 2; OD, opacity density score; Ctrl, control; IL, interleukin; Dex, dexamethasone; GR, glucocorticoid receptor; HDAC2, histone deacetylase 2.
